# An Introduction to AI for Clinicians: Tutorial

**DOI:** 10.2196/85266

**Published:** 2026-03-30

**Authors:** Stephen B Lee, Alexis B Carter, Muhammad Hamis Haider, Seok-Bum Ko

**Affiliations:** 1 Division of Infectious Diseases, University of Saskatchewan, 1440-14th Avenue, Regina General Hospital, 2nd Floor Medical Office Wing, ID Clinic, Regina, SK, S4P 0W5, Canada, 1 3067664247; 2 Department of Pathology and Laboratory Medicine, Emory University, Atlanta, GA, United States; 3 Department of Electrical and Computer Engineering, University of Saskatchewan, Saskatoon, SK, Canada

**Keywords:** medical education, machine learning, artificial intelligence, AI, deep learning

## Abstract

Artificial intelligence (AI) is already fundamentally changing society, with medicine being no exception. AI will impact how we practice, how hospitals operate, and even the practice of medicine itself. The use of AI-based products has already begun, with examples including AI scribes and large language models such as ChatGPT. Work is ongoing to produce models that have specific functions within medicine, such as kidney injury prediction. However, transformative foundational work, such as AlphaFold (for protein structure prediction), also promises to completely change the way we approach medicine. Therefore, clinicians must develop a clear understanding of AI, not as an optional skill, but as a core competency of modern medical practice. This paper serves as a tutorial to guide medical professionals through the basic principles of AI. It will teach clinicians how to build a mental scaffold to understand and springboard into AI. The core parts of this paper are organized in steps, with additional relevant topics addressed in modules at the end of the paper. The core steps are meant to be read sequentially. To prepare the reader for the rest of the paper, this tutorial will first introduce what AI is and then cover some basic definitions needed to understand other concepts. The reader will then be ready to understand what deep learning is and the difference between supervised and unsupervised learning. Finally, the reader will go through how deep learning models learn. Separate modules on safety and clinical applications are also included. This tutorial is relevant to clinicians at all levels but may be particularly useful for practicing clinicians who are encountering AI tools integrated into their practices without previous formal education in the field. Users of this tutorial can refer to specific sections or read the entire paper.

## Step 1: Understanding What Artificial Intelligence Is

**Figure 1.**
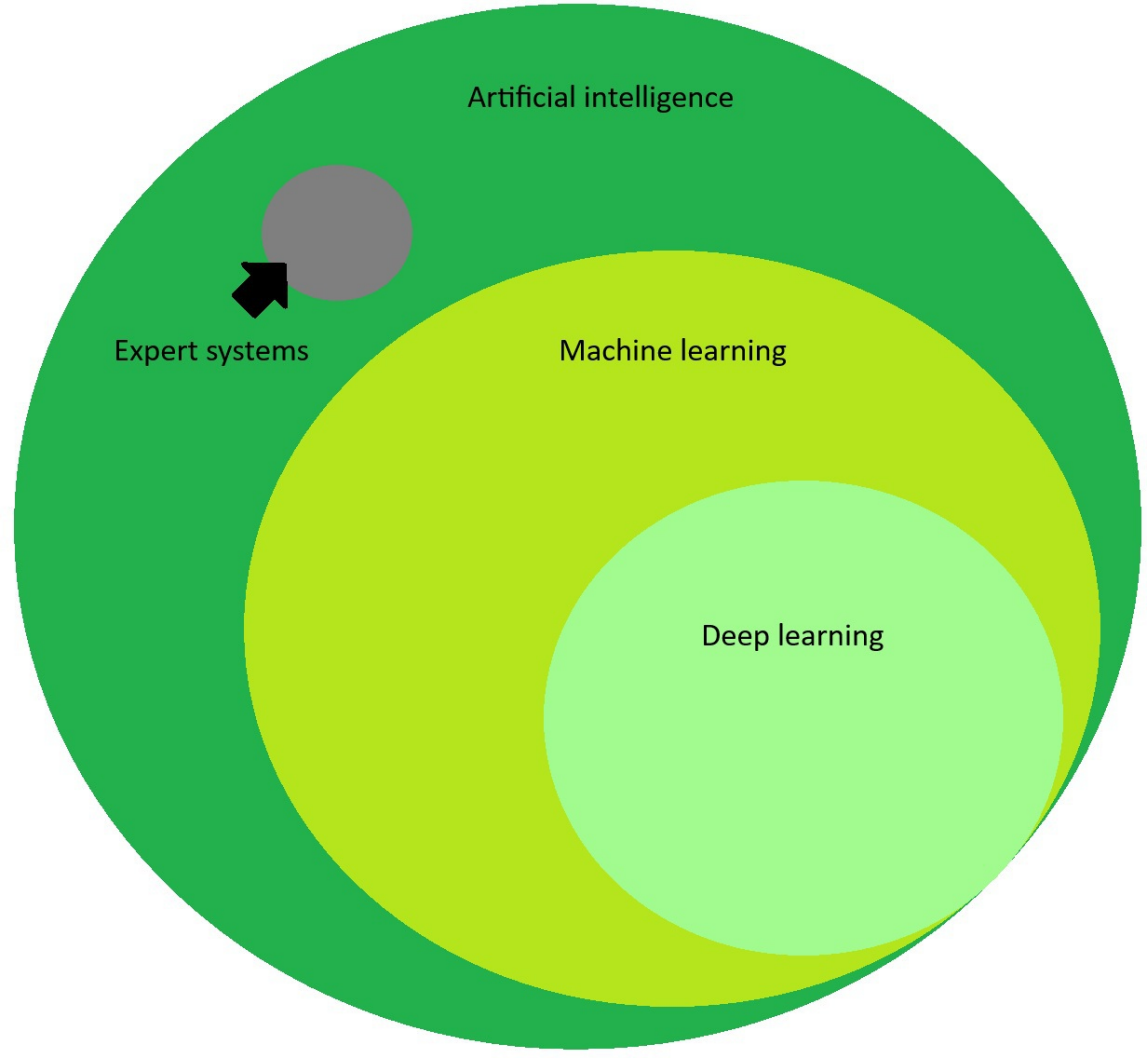
Venn diagram of artificial intelligence terminology.

Before delving into the concepts underlying artificial intelligence (AI), it is important to understand what AI means. AI is a broad term that generally refers to computer systems that can perform complex tasks historically associated with humans, such as human learning, comprehension, problem-solving, decision-making, creativity, and autonomy [1]. In computer science, AI algorithms encompass a variety of methodologies. Most AI in the modern era is machine learning (ML) and even more specifically, deep learning (DL; Figure 1). While all ML is considered AI, not all AI is ML.

Expert systems are an older paradigm of AI in which a subject matter expert’s knowledge is hardcoded into intricate rule-based algorithms to simulate human decision-making (eg, “if x condition, then y result”). Well-known examples include the MYCIN system, designed by Edward Shortliffe in 1974 to predict antibiotic choice [2], and traditional chess AI systems. Even famous examples such as Deep Blue (IBM Corp) relied on hardcoded knowledge [3]. Expert systems and these hardcoded methodologies have been largely abandoned in the modern era due to the incredible effort required to develop them and the brittleness and inflexibility of the resultant systems. Limited examples of expert systems also exist in health care, such as older sepsis and drug interaction clinical decision support systems. AI is primarily ML currently. Rather than relying on hardcoding from experts, ML algorithms are trained to learn relationships and patterns in data. The terms DL and ML are often used interchangeably; however, it is worthwhile mentioning that DL is really a subset of ML. There are many types of ML that are not DL (eg, k-means clustering and decision trees). DL is discussed in depth in a following section.

## Step 2: Understanding Basic Definitions

Some level-setting is required to understand ML algorithms and models. ML *algorithms* are tools (eg, logistic regression) that are trained on data to create an ML *model*. ML models that are used for classification are frequently referred to as *classifiers*. The quality of the data used to train an algorithm is critical to the performance of the model. Each record or event in the dataset is referred to as an *instance*. Each aspect of an instance (eg, color, duration, and test result) used to train a model is known as a *feature*. In a simple set of data, instances would be rows in your data spreadsheet, while features would be the column headers. Many ML datasets can have thousands of instances and hundreds of features.

A *label* is the information on a particular feature for a certain instance in the dataset (eg, “red” for a feature of “color,” “30 minutes” for a feature of “duration,” and “34 mg/dL” for a feature of “glucose level”). A label generally refers to information that has been applied by a human or another algorithm based on manual or traditional analysis of the data and represents a classification, categorization, ranking, or answer to a question. For example, in a dataset of inpatients hospitalized for at least 30 days with a feature of “patient developed a hospital-acquired infection during admission,” the label would be “positive” if an infection was acquired and “negative” if none occurred, bearing in mind that the application of positive or negative would have to be done by a human who was analyzing the patient’s data.

Of note is that data that are not manually labeled are simply referred to as data. One may also choose to use only a subset of the data in a dataset for training.

## Step 3: Differentiating Between Supervised and Unsupervised ML

A core initial concept is understanding the difference between 2 major categories of ML. In general, ML can be classified into supervised and unsupervised learning, although there are other categories or approaches such as reinforcement and transfer learning.

**Figure 2.**
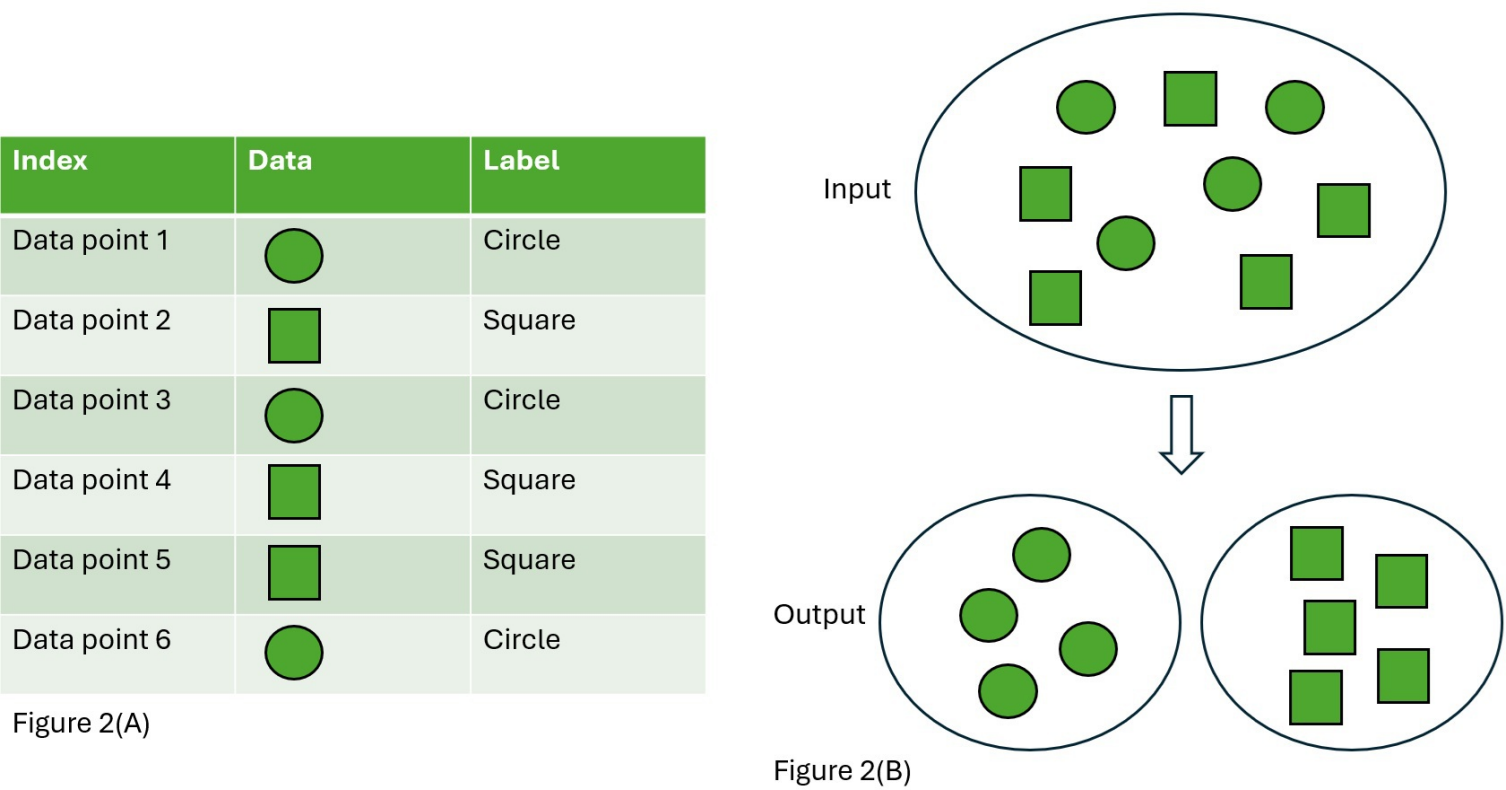
Illustration of (A) supervised and (B) unsupervised learning.

In *supervised learning*, data that have been labeled are fed into the ML algorithm for training. Once the algorithm has been trained, it is called a *model*. One of the greatest challenges in developing a high-quality model is being able to obtain a substantial amount of data (ie, usually thousands of instances) that have been accurately labeled. An example of supervised learning would be an algorithm trained on a dataset of chest X-rays and corresponding diagnoses [4-6]. In *unsupervised learning*, unlabeled data are fed into an algorithm, and the algorithm discovers relationships and groupings for itself. This difference is illustrated in Figure 2. An example of unsupervised learning is work in which an algorithm identified distinct clusters or groups of patients who had COVID-19 [7,8].

In Figure 2A, the shapes are labeled as circles and squares by a human. The dataset is fed into the algorithm, and through these labels, the machine learns which features contribute most to classifying the shapes. In Figure 2B, the machine is fed raw data without any labels. Through exploration and differences detected in the data among its features, the model learns that there are potentially 2 different categories of objects. Of note is that the model may not inherently recognize them as a circle or a square but rather as 2 distinct categories of objects.

Other forms of learning also exist, such as *reinforcement learning*. In reinforcement learning, an algorithm experiences an environment and takes an action. On the basis of this action, the model is given a reward or a punishment as feedback. The algorithm learns which patterns result in rewards vs punishments and adjusts its behavior accordingly. Furthermore, supervised and unsupervised learning exist on a continuum, and some forms of learning are a mixture of both (ie, some instances are labeled, while others are not). A full description of these concepts is beyond the scope of this paper.

Transfer learning is another method in which an algorithm is first trained on a very large but nonspecific set of data for the desired outcome and then uses the learned patterns on a smaller but more specific set of data to refine the algorithm into a model. Transfer learning is often used where high-quality labeled datasets specific to the subject area are limited, whereas broader, nonspecific datasets are more plentiful.

## Step 4: Defining DL and Neural Networks

A subcategory of ML is DL. This branch of ML is heavily focused on neural networks. *Neural networks* were first described in the mid-20th century [9,10] and were designed to emulate neural processes in the human nervous system. While foundational work has been ongoing for decades in the field [11-13], much early work was constrained by hardware and data availability. The emergence of powerful parallel computing, called graphics processing units; platforms to leverage graphics processing units; and the availability of large datasets have helped overcome these barriers [14].

Artificial nodes, called *neurons*, are connected in layers to form a network. Data are put into the input layer of the network; the network processes the data through one to many hidden layers and then provides results in the output layer.

DL specifically refers to ML done on neural networks with many layers (thus the term “deep”). There is no exact number of layers that is generally agreed upon as a threshold. However, common examples such as ResNet (Microsoft) and the architecture underlying ChatGPT (OpenAI) can contain a hundred layers and billions of parameters [15,16].

## Step 5a: Walk-Through on How ML Algorithms Work

Next, this tutorial will lay out the process by which ML algorithms work, describe the process with an example, and then define other key terms. To learn how to properly predict, classify, or rank instances in supervised learning, an algorithm analyzes the data to determine which features contribute the most to the data labels. This learning process is called *training*. During training, the machine learns to adjust internal parameters, called *weights*, to produce the desired outputs. These weights correspond to individual neurons. This occurs through minimizing loss functions, in which the gap between the observed and expected prediction is minimized.

For example, consider a model designed to estimate the risk of candidiasis in hospitalized patients. The model may learn that features such as intensive care admission, fever, abdominal symptoms, or a normal white blood cell count are associated with either a higher or lower risk of candidiasis. Features will often be mapped in a complex fashion across a series of neurons and layers. Modification of the weights applied to each of these features influences the resulting model’s output through complex interactions across many layers of analysis, allowing the model to learn patterns that relate input features to the target outcome. In simpler forms of ML, it is sometimes possible to determine which features most greatly contribute to an outcome; however, in DL, this is often difficult because of the number of nodes and layers involved. Therefore, in DL, the change in importance may not be directly interpretable.

Datasets are often large and contain many features, some of which logically have nothing to do with the outcome being examined. If the data used to train an algorithm contain these completely unrelated variables, the algorithm may make nonsensical associations, which can later result in spuriously wrong results. For example, it may associate the color of a hospital gown and/or patient’s sandwich preference with overall length of stay. However, if there are different colors of hospital gowns or menu choices for patients in intensive care units vs regular floors that are not captured in the data, then the model may miss the confounding variable completely. These errors in model development are hard to detect in all ML and even harder in DL.

## Step 5b: Walk-Through of Basic ML Mathematics Using an Example—Loss Function

To illustrate the mathematics of ML training, we can use an extremely simplified example with a continuous variable in supervised learning. It is noted that most modern DL requires massive sets of data. While specifics may change in other forms of ML, this example will illustrate general concepts. In this example, the dataset includes the trough levels of a nephrotoxic drug and the resultant measured (true) estimated glomerular filtration rate (eGFR) in patients. The measured eGFR is the labeled data in this supervised ML. An appropriate ML algorithm is selected that attempts to predict the eGFR based on drug levels (Table 1).

**Table 1.** Sample data for a model predicting nephrotoxin toxicity.

Nephrotoxin level (mg/L)	True eGFR^a^ (y; mL/min/1.73 m^2^)	Machine learning model’s predicted eGFR (ŷ; mL/min/1.73 m^2^)
10	100	95
13	95	92
15	100	90
20	90	85
25	80	80
30	65	75
40	55	65

a eGFR: estimated glomerular filtration rate.

**Figure 3.**
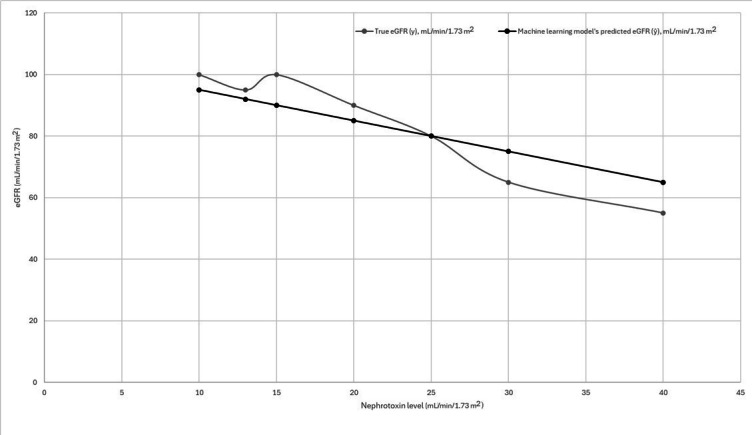
Sample data for a model predicting nephrotoxin level. eGFR: estimated glomerular filtration rate.

The model produces a prediction (ŷ) based on the nephrotoxin level (x). The algorithm compares the predicted eGFR (ŷ) to the real measured value of eGFR (y). Graphically, the predicted values of eGFR (the orange line in Figure 3) are plotted against the true values (the blue line). To determine the pattern between the nephrotoxin level (x) and the measured value of eGFR (y), the algorithm calculates the loss function. Differences might appear small in most regions but noticeable in some. To capture a model’s performance across all data points, we use this loss function, a mathematical function that quantifies the total error between predicted and observed values.

By convention, updates are made to the loss function after each batch in training. The model then adjusts the weights and tries again to determine, across all the instances in the dataset, which set of weights will produce the lowest loss function on average for the training data. With high-quality data that represent the full spectrum of possible cases that the model could be given, this helps ensure that the model is trained to perform well on average and does not result in spuriously wrong predictions. The specifics of how the algorithm calculates loss functions vary between algorithm types, with different functions being optimized for different tasks. For instance, the mean squared error, a common loss function, squares the difference between ŷ and y. By squaring the difference, it ensures negative loss values do not cancel positive values when summed, and it helps penalize severely wrong predictions.

In a model that performs well when it is fed new data (ie, generalizes well), ŷ and y will be similar for any new patient’s nephrotoxin level fed into it. Models that do not generalize well may make large, nonsensical errors in prediction for a small subset of patients with a few specific differences in data points. This is why training a model on high-quality data that accurately represent the types of data it may encounter after deployment is critical.

## Step 6: Understanding the Concepts of Backpropagation and Gradient Descent

**Figure 4.**
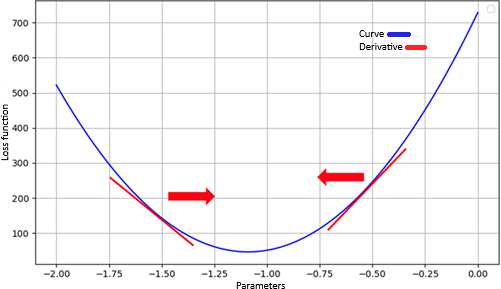
Graphical illustrations of concepts.

Another set of key concepts to understand is backpropagation and gradient descent. In DL, a model modifies the weights of specific neurons within a neural network to create its predictions. When data are input, they forward pass through the network. The initial run creates random weights, and the prediction ability of the initial model is likely poor. However, after this forward pass, the model evaluates its resultant loss function and attempts to minimize the loss through a process called gradient descent. In gradient descent, we assign a learning rate, which increments our point along the curve of the loss function (Figure 4). If the model discovers that the loss is increasing, it will move backward to decrease the loss. Conversely, if it discovers that the loss is decreasing, it will continue moving in that direction. In doing so, it eventually seeks out the local minima, thus minimizing the loss function and improving the model’s predictive capacity.

Mathematically, gradient descent can be expressed as$w=w-\eta \left(\frac{dL}{dw}\right)$. Remember that the derivative $\left(\frac{dL}{dw}\right)$ of the loss function gives the gradient at any given point. This allows the model to know if the loss is increasing or decreasing. The learning rate (η) tells the model how much to move in each direction. Recall that the loss function quantifies the difference between the predicted and observed values, with the goal of identifying the lowest point and, thus, minimizing this difference. These concepts are illustrated again visually in Figure 4. The mean squared error of our example model’s simple line (Figure 3) is a parabola, creating an easy visualization of gradient descent for illustration purposes. When η is set at too large a value, it is possible that the model will jump very far back, such that it misses the local minima. Conversely, too small a value of η may result in such minimal movement that the minima is never reached.

During the training process, to specifically update the gradient, the model will undergo a process called backpropagation. The trainer algorithm will go through the neural network and alter the weights of neurons with the intention of decreasing loss. This process ultimately results in better prediction ability of the ML model.

## Module 1: A Brief Explanation of AI Explainability and Safety

As AI becomes significantly more powerful and integrated into society, various risks and errors have been observed. A brief discussion of safety is outlined here, and there are many other publications available that dive into each of these in detail.

How an ML model determines its output (ie, resultant prediction or result given to a user) from input is often unclear. This refers to the “black-box” nature of all ML but particularly of DL and neural networks. A field called explainable AI has emerged and attempts to not only better understand how models make their predictions but also add components to the ML model that require the model to explain how it arrived at its result (ie, which features had the most impact) [17].

For all AI, especially in medicine, it is imperative that model performance be checked for variations in outcomes that may indicate that human bias in the data has been promulgated or exacerbated by the model. AI algorithms are exquisitely sensitive pattern detection tools. As such, they can detect slight variations in data that resulted from human prejudice and bias. Worse, they can promulgate such bias into the model. Therefore, it is imperative that models be checked for differences in results that can be attributed to race, gender, socioeconomic status, religion, etc, as the presence of these elements in a model will cause the model to produce inaccurate results in certain groups of patients. Concrete examples include models having difficulty diagnosing dermatological conditions in those with darker skin tones [18], and a software program that accidentally referred White patients over African American patients to receive special care [19]. In the latter example, while the goal was to ensure patients received the care required, the algorithm was designed to predict whose care would cost more money, and it was found that less money was spent on African American patients despite having the same level of need [19]. Furthermore, a study found that an AI system could learn to predict ethnicity from radiographs alone [6]. There are now tools that assist in the detection of these elements, and models that include these elements need to be retrained on optimized data or otherwise mitigated to ensure the best care for all patients. Finally, risks associated with the infrastructure of AI also exist. As many models use a cloud-based model for computation and some public commercial large language models (LLMs) use input data to retrain models, clinicians need to be aware of where sensitive data are being sent and stored.

Now that the use of AI is rapidly becoming more pervasive, it is reasonable to think that the degree to which AI is set to perform autonomously (ie, without a human in the middle) will increase. Some publications have discussed safety measures that are required to mitigate risks as AI becomes more autonomous and integrated into systems. These risks range from tangible risks today to theoretical risks with more powerful models. Experts have classified these risks into 4 categories: misuse, misalignment, mistakes, and structural risks. Misuse occurs when users intentionally instruct an AI tool to behave in harmful ways (ie, the user is an adversary). Misalignment occurs when AI systems knowingly act against human intent (ie, the AI is an adversary). This includes intentional deception by AI. Mistakes occur when AI systems produce incorrect outputs without intentional wrongdoing, often because real-world data are complex and influenced by many contributing factors. Finally, structural risks may emerge whereby pervasive AI systems integrated into society cause harm through the actions of multiple independent agents in a multifactorial, multiagent fashion [20].

A substantial body of ongoing research in AI focuses on ensuring and improving AI safety. For instance, studies focus on the effects of adversarial attacks on models to understand safety. Developers also attempt to build safeguards into models and use red teaming, where security professionals attempt to simulate attacks to determine robustness [4,18]. Equally important are efforts to create nuanced and well-informed regulations and guidelines for development [18,21-26].

## Module 2: Contemporary Clinical Applications of AI in Medicine

Health care is one of the most promising industries for AI. While disruptive-level work is underway, such as the ability to understand protein folding [27], AI has numerous applications currently being used routinely in health care.

Documentation is recognized to often be excessive and contributory to physician burnout, with an American Medical Informatics Association survey finding that 73.26% of health care professionals believed the time spent was inappropriate, 77.42% reported after-hours work related to documentation, and 74.83% believed that documentation impedes patient care [28]. Canadian data show similar findings, with physicians spending excessive amounts of time on administrative tasks that result in burnout [29,30].

AI scribes are tools that can ambiently listen to patient interactions and automatically generate notes for physicians, reducing the administrative burden for physicians. Numerous companies have created offerings; however, in general, scribes use LLMs, models based on the transformer architecture (which uses an attention mechanism to process preceding information and learn relationships among them) [31,32]. Within the context of scribes, the LLM uses this mechanism of self-attention to help generate logical text. Thus, the same limitations of transformers and LLMs carry forward onto AI scribes. For example, in many LLMs, hallucinations are a concern, which are theorized to arise because of algorithms being rewarded for correct responses, thereby making guessing a more advantageous response than acknowledging uncertainty. Health care providers must be aware of these limitations of AI scribes and how they may potentially arise.

LLMs have also been used by vendors such as Epic to automatically extract information out of existing notes, such as creating discharge summaries, reading radiology reports, providing summaries, preparing tasks based on the note being created, and providing insights [33]. They have also been used as chatbots to act in clerical roles and as search engines for medical knowledge [34,35]. While LLMs such as ChatGPT (OpenAI), Claude (Anthropic), and Gemini (Google) are commonly used by the general public, OpenEvidence (OpenEvidence LLC) is trained specifically on medical literature, reducing errors and hallucinations [36]. Models intended for use in research and science also exist, such as Perplexity (Perplexity AI) and Elicit (Elicit Research) [36,37]. Open source, medically-focused models without interfaces also exist, such as MedGemma (Google) [38].

Under the broader definition discussed in this paper, AI has been used in diagnostics for decades using simple rule-based algorithms. However, more recently, ML-DL–based approaches have begun to gain traction due to improved performance. Areas that have shown promise for ML-DL performance are radiology and pathology [39,40]. In radiology, a DL software such as CINA-iPE (Avicenna.AI) has shown promise in detecting pulmonary embolisms [41]. In pathology, ML has improved rapid patient diagnostics based on DNA methylation markers [42]. Sepsis and cardiac arrest prediction have been an ongoing area of work [43,44]. While results from this work are promising, due in part to interpretability, accuracy, and the potential for uncovering clinically irrelevant abnormalities (“incidentalomas”), it is unclear how clinicians should react to findings [45]. The volume of AI tool uptake is increasing, and AI will inevitably impact the workflow of clinicians in the near future. AI could also support hospital infrastructure [46].

Despite its potential benefit, AI integration into health care is still an evolving landscape. Regulation and guidance remain an important area of evolving work for health care AI, with numerous national and international bodies producing frameworks and guidelines for AI use [47-49]. Important questions that remain under debate include the responsibility for AI errors, with many institutions holding physicians ultimately accountable for clinical decisions, how to mitigate bias in health care AI propagated by inherent bias in training data, and the importance of privacy [6,18,19,50]. Furthermore, many studies have indicated that implementation into workflows can also be challenging, due to a lack of awareness and engagement by both patients and health care professionals, as well as logistical implementation challenges inherent to any health technology [51].

## Future Directions

AI promises to be one of the most critical revolutions of human society, arguably on par with the industrial or even agricultural revolutions. AI will impact every area of human society, including health care.

While current implementations such as LLMs, predictive models such as convolutional neural networks, and the tools they have created (eg, AI scribes and ChatGPT) are influential, most of the society-changing work is being dedicated to creating artificial general intelligence and artificial superintelligence. While the exact definition of these terms, and even their possibility, is a matter of contention [52], they generally refer to AI systems that are either as intelligent as or more intelligent than human beings across a wide array of domains and tasks.

In achieving this goal, AI will have implications on the role of humans in a post–artificial general intelligence society. While there are critical concerns about human displacement, there is also a potential for creating abundance, reducing scarcity, and an ability to supercharge scientific discovery [53].

Due to its importance, the authors believe it is important that clinicians receive structured educational content on the topic. Leaders could consider integrating formal foundational AI teachings into medical school curricula and, then in later years, providing a chance to discuss its implications and applications in health care. These sessions could also be incorporated into postgraduate education and into continuing medical education sessions offered by workplaces.

## Abbreviations

AI: artificial intelligence
DL: deep learning
eGFR: estimated glomerular filtration rate
LLM: large language model
ML: machine learning
